# A Based Bayesian Wavelet Thresholding Method to Enhance Nuclear Imaging

**DOI:** 10.1155/2009/506120

**Published:** 2009-03-26

**Authors:** Nawrès Khlifa, Najla Gribaa, Imen Mbazaa, Kamel Hamruoni

**Affiliations:** Research Unit of Signal Processing, Image Processing and Pattern Recognition, National Engineering School of Tunis, 1002 Tunis, Tunisia

## Abstract

Nuclear images are very often used to study the functionality of some organs. Unfortunately, these images have bad contrast, a weak resolution, and present fluctuations due to the radioactivity disintegration. To enhance their quality, physicians have to increase the quantity of the injected radioactive material and the acquisition time. In this paper, we propose an alternative solution. It consists in a software framework that enhances nuclear image quality and reduces statistical fluctuations. Since these images are modeled as the realization of a Poisson process, we propose a new framework that performs variance stabilizing of the Poisson process before applying an adapted Bayesian wavelet shrinkage. The proposed method has been applied on real images, and it has proved its performance.

## 1. Introduction

Nuclear medicine provides morphological and anatomical functional information, which represents one of its important advantages. Images are obtained by detecting the emitted radioactivity of an isotope previously injected to the patient. The amount of the radioactive substance is carefully selected in order to reduce the acquisition time while ensuring an accurate test [1].

In nuclear images (called also scintigraphic images), the pixel value is proportional to the real radioactivity emitted by the explored organ which reflects its functionality level. In practice, it is difficult to establish this proportionality because of the acquisition system and the statistical fluctuations. These values follow a statistical Poisson distribution due to the random nature of radioactive disintegration [2]. Images are then modeled as the realization of a Poisson process.

In this work, we propose a soft framework that enhances nuclear image quality and reduces statistical fluctuations. This framework performs variance stabilization of the Poisson process before applying an adapted Bayesian wavelet shrinkage.

In the remaining sections of the paper, we first present some background on nuclear image degradation. In the third section, we present an overview of some important related work. In the fourth section, we describe the proposed method in detail. Some experimental results and discussion are presented in the fifth section. Finally, the main conclusions are summarized in Section 6.

## 2. Noise in Nuclear Images

Nuclear images are modeled as a Poisson process [3]. Because of the randomness of the radioactive decay, the number of photons detected during a time interval is not constant and follows the statistical Poisson distribution given by

(1) $$p(n)=\frac{N^{n}e^{-N}}{n!}.$$

*N* is the mean value of the distribution. This probability is maximum for *n* = *N*.

These statistical variations involve the Poisson noise in scintigraphic images [2]. Since the variance *σ* ^2^ of a Poisson distribution is equal to its mean value, the standard deviation *σ* of the distribution is equal to $\sqrt{N}$.

In a pixel (*i*, *j*), the average *N* of the Poisson distribution is given by

(2) N(i,j)=A⋅τ⋅K(i,j),

where *A* is the injected activity, *τ* is the acquisition time and *K*(*i*, *j*) is the “ideal” image which can be obtained when there is no radioactive emission [2].

The importance of statistical variations is quantified by the noise to signal ratio given by

(3) $$\begin{array}{ll} R(i,j) & =\frac{\begin{array}{cc} \text{standard}\;\;\text{deviation} & \end{array}}{\text{average}}\\ & =\frac{\sqrt{N(i,j)}}{N(i,j)}=\frac{1}{\sqrt{N(i,j)}}=\frac{1}{\sqrt{A\cdot \tau \cdot K(i,j)}}. \end{array}$$

To decrease this ratio, we must

- increase the acquisition time *τ* (if the body dynamics allow it). Scintigraphic images quality strongly depends on the acquisition time. This is a real problem and a source of daily dilemmas for nuclear physicians;
- inject more activity *A* while respecting the dosimetric constraints;
- use gamma cameras with a powerful detector or with multidetectors. This can generate technical and financial problems;
- increase the elementary acquisition surface. This can reduce the image resolution.

Considering all these difficulties, researchers try to find software solutions based on image processing. These solutions aim to

- suppress fluctuations due to counting statistics to allow reliable quantification of diagnostic parameters and to allow a good reconstruction of PET and SPECT images;
- improve the detection of the low-size lesions;
- enhance image contrast to facilitate the interpretation;
- increase regions homogeneity to assist the localization of regions of interest [1].

However, nuclear image processing should preserve region boundaries and small details and should not generate artifacts.

## 3. Related Works

The first attempt to enhance nuclear images started with the setup of the first gamma cameras. Until now and in spite of the notable improvement of gamma cameras [3], many researchers focus on developing solutions to remove noise from scintigraphic images. Some works consider the general framework of restoration while others focus on the noise removing task.

The denoising—or the nonparametric regression in statistical mathematics—is nowadays a powerful tool in signal and image processing. Its main goal is to recover a component corrupted by noise without using any parametric model. In the beginning, linear and nonlinear filters are used, but their immediate consequence is contrast degradation and details smoothing [3]. To overcome this limitation, several nonstationary filters have been proposed [4], but they are not used in daily practice. Probably, this is due to the artificial appearance of the processed images; their texture is relatively different from that of the original images [4].

Actually, denoising using wavelets proves its ability to satisfy the compromise between smoothing and conserving important features. The observed data are modeled as a signal embedded in noise. When the noise is additive and Gaussian, the denoising problem becomes how to determine the optimal wavelet basis that concentrates the signal energy in a few coefficients and thresholds the noisy ones.

**Figure 1:**
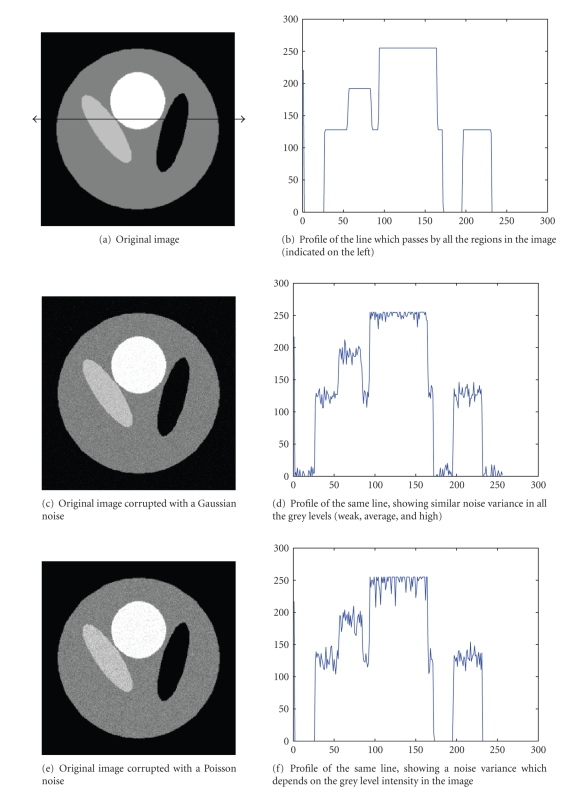
Illustration of the difference between a Gaussian and a Poisson noise.

However, in several experimental domains, especially those based on techniques where the detection involves a counting process, the data is modeled as a Poisson process (which is the case for scintigraphic images). In this context, several techniques where considered in order to recover the underlying intensity structure. Unlike the Gaussian noise (which is independent), the Poisson noise depends on the image intensities (Figure 1 simulates the difference between the Gaussian and the Poisson noise). Consequently, the wavelet shrinkage is not suitable for this context.

A straightforward method to deal with this problem is to introduce a preprocessing normalizing step such as the Anscombe [5] or the Fisz transform [6]. The noisy image is then transformed into an image contaminated with approximately Gaussian noise with a constant variance. Thus, this variance-stabilizing operation leads to estimate the underlying intensity function by applying one of the many denoising procedures already designed for Gaussian noise.

In this context, several proposed Bayesian estimators were more efficient than classical ones. In the Bayesian paradigm, a prior distribution is placed on wavelet details coefficients. So, the estimated image is obtained by applying the appropriate Bayesian rule on these detail coefficients. For the existing Bayesian approaches, we can distinguish univariate and multivariate density estimation both achieving interesting results in practice [7, 8]. In addition, referring to the comparison of different approaches provided by Kirkove [9] and by Besbeas [10], we can conclude that the Bayesian estimators perform interesting results [7].

Another approach consists of dealing with the simple Haar transform since it is the most suitable basis for Poisson-like models [11]. This method was introduced by Kolaczyk [12], Charles and Rasson [13], Willett and Nowak [14]. It is based on the Haar wavelet coefficients shrinkage of the original counts (without any preprocessing) using scale-dependent thresholds.

## 4. Proposed Framework

Scintigraphic images are corrupted by a Poisson noise. In this framework, we will first start by a normalizing step; the choice of the stabilization transform will be explained in the first subsection. The resulting image can be considered as if the noise was Gaussian. Then, we will apply a modification on the Bayesian shrinkage rule known in the literature to exhibit good results as for white Gaussian noise. The method efficiency is evaluated on synthetic and real images.

We begin by presenting some theoretical aspects needed to understand the proposed method.

### 4.1. Phase 1: The Poisson Distribution Normalization

This step can be achieved by the Anscombe or the Fisz transform. The Fisz transform is the most recent one. It has been extended to the 2D case by Fadili et al. [15]. This method is based on the asymptotic normality of the Haar wavelet and scale coefficients. In their work, some asymptotic results such as normality and decorrelation of the transformed samples are extended to the 2D case.

In our works, published in [16, 17], we have coupled the Fisz transform to a shrinkage wavelet estimator. The method performed good results only in the very low-count setting. This can be explained by examining the theoretical aspects of the Fisz transform. In fact, it can achieve good results in both smooth and piecewise constant intensities. Thesis constraints cannot be verified in scintigraphic images. So, we propose to use the Anscombe transform which can be used in all count setting.

The Anscombe transform consists of 4 steps.

**Step 1**

Compute the Anscombe transform:

(4) y=Ax,

where *y* is the underlying intensity function and *x* is the original image, so, for every observed count *xi*, $yi=2x\sqrt{xi+(3/8)}$ is defined.

**Step 2**

The resulting image *y* can consequently be modeled as

(5) y=Ax+ε,

where *ε* is a Gaussian white noise with constant variance.

**Step 3**

Apply a denoising process to the obtained image contaminated by a quietly Gaussian white noise.

**Step 4**

Apply the inverse Anscombe transform to determine the underlying intensity function estimation x^.

### 4.2. Phase 2: The Bayesian Wavelet Shrinkage

In literature, several proposed Bayesian estimators were proved to be more efficient than classical ones. In fact, referring to recent works published in 2004 and in 2007, a comparison between different approaches applied on different images corrupted with Gaussian noise was made, and it was proved that the Bayesian estimators perform interesting results.

In this framework, we will make use of the Bayesian technique developed in [7], and it is considered one of the most important works in this field. In fact, it was used by many researchers to develop their own estimator.

In this section, we present first the original Bayesian estimator, then our attempt to adapt this estimator to scintigraphic images.

#### 4.2.1. Bayesian Threshold

The Bayesian thresholding is proved to be one of the most efficient denoising formalism. It was shown in [7] that, for a white Gaussian noise, the threshold given by (6) is optimal:

(6) $$T_{\beta }(\sigma_{X})=\frac{\sigma^{2}}{\sigma_{X}},$$

where *σ* ^2^ is the noise variance.

Since the noise is “iid” type (independent and identically distributed), we can write

(7) $$\sigma_{Y}^{2}=\sigma_{X}^{2}+\sigma^{2},$$

where *σ*_*Y*_^2^ is the estimated variance of the observed image. The estimated variance of signal *σ*_*X*_^2^ is then deduced by

(8) $$\sigma_{X}=\sqrt{\max\;(\sigma_{Y}^{2}-\sigma^{2},0).}$$

A robust estimator of the noise variance is obtained by

(9) $$\sigma =\frac{M}{0.6745},$$

where *M* is the median value of the absolute wavelet coefficients in the first decomposition level.

It was proved that this threshold value is optimal, assuming that wavelet coefficients are Generalized Gaussian Distribution (GGD). The GGD depends on a parameter *β* called shape factor [7]. It was shown that a value of *β* belonging between 0.5 and 1 can model various subbands of a large set of natural images. In all works using the Bayesian threshold, this parameter is fixed to 1 to simplify the problem. And it is suggested to integrate this shape factor to compute the threshold value [7].

Tow major modifications are applied to the Bayesian threshold. They will be presented in Section 4.2.2:

- the search for a shape factor *β* that better adapts the scintigraphic images;
- the use of the undecimated wavelet transform instead of the decimated one.

#### 4.2.2. Subbands Modeling the Scintigraphic Images

**Figure 2:**
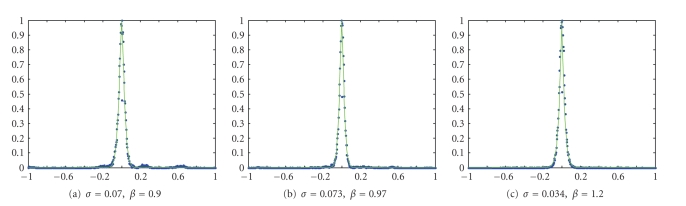
Example of the subband modeling of, respectively, the horizontal, the vertical, and the diagonal subbands (in blue the coefficients distribution, in green the GGD with the given parameters).

To better estimate the *β* value for the scintigraphic images, we have used 100 images; each one has been decomposed in 3 levels. We obtained then more than 1000 subbands to be modeled. For each subband, we seek the *β* value that better approximates the coefficients distribution by a GGD. An example of results is given in Figure 2.

**Figure 3:**
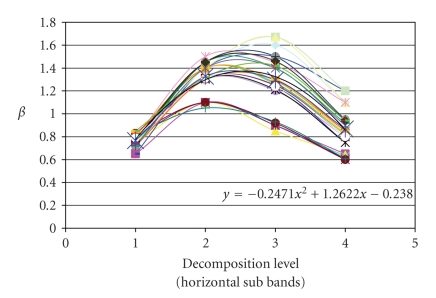
*β* variation in horizontal subbands of a set of scintigraphic images (in black the polynomial *y* that approximates the average variation).

Statistical study on the *β* variation obtained in the set of 100 images is given in Table 1. According to this table, we notice that in more than 58% of the situations, the value of *β* is out of the range [0.5,1], and in more than 52%, the value of *β* is in the range [1,1.5]. However, this value estimation for each image appears as a difficult task. To simplify the problem, we proposed to compute the average of the *β* values, and to plot its variation against the decomposition level (according to each direction). This variation can be approached by a polynomial of degree 2. An example of *β* variation is given in Figure 3. The three found polynomials are given in the expressions (10) (corresponding respectively to vertical, horizontal, and diagonal direction)

(10) $$\begin{array}{ll} \beta_{v} & =-0.2335X^{2}+1.2187X-0.1698,\\ \beta_{h} & =-0.2471X^{2}+1.2622X-0.238,\\ \beta_{d} & =-0.1914X^{2}+1.124X-0.2949, \end{array}$$

where *X* indicates the decomposition level.

To compute the threshold value, we propose to integrate the *β* value according to expression (11). This will be done for each direction (horizontal, vertical, and diagonal). That is to say, for each direction, a threshold is calculated. This threshold varies from a level of decomposition to another. It is important to underline, that this value, is optimal in the PSNR sense.

(11) $$T_{\text{dir}}=\exp(\beta_{\text{dir}})\log\;(n)\frac{\sigma^{2}}{\sigma_{X}},$$

where *β*_dir_ is the directional value of *β* calculated according to the expression (10), and *n* is the image size.

#### 4.2.3. Use the Undecimated Wavelet Transform

It is known that thresholding in orthogonal wavelet domain produces observable artifacts (such as oscillations due to the Gibbs phenomenon near contours). To reduce this disturbed phenomenon, Coiffman and Donoho proposed the translation-invariant denoising algorithm. The discussion is made in 1D [18]; an extension to 2D is exposed in [7]. The translation invariant algorithm can be considered as equivalent to thresholding in undecimated bases decomposition. This decomposition is a redundant representation of the image, and then the coefficients are correlated. The thresholding approach is not suitable since the distribution is not iid.

To improve the results, we propose to extend our framework to process iid distribution. To do so, we propose to separate the coefficients resulting from the redundant decomposition in four sets of not correlated coefficients. For each direction, we will rearrange the coefficients (*i*, *j*) in four sets, according to

(12) $$\begin{array}{c} \{Y[2i,2j]\},\{Y[2i,2j+1]\},\\ \{Y[2i+1,2j]\},\{Y[2i+1,2j+1]\}. \end{array}$$

Since the coefficients in each set are not correlated, the thresholding algorithm can be applied for each set.

## 5. Results and Discussion

In order to evaluate our method, we have collected two sets of data.

- The first set contains planar cardiac images acquired with an ascending total counting. Images correspond to a ventriculography whose acquisition is synchronized with the ECG. We have increased the number of superposed cycles. Consequently, the acquisition time *τ* and the total counting have been increased. This reduces the noise in image according to (3). We can then assume that the same image acquired with an ascendant time is the reference image, and consequently we can compute the PSNR value.
- The second set contains a set of scintigraphic images of other organs like the bone, the thyroid, and so forth.

To evaluate the method performance, we used two types of evaluation tests.

(1) The first one consists of comparing the proposed framework with other methods well known in this field. The chosen methods are

- the Hanning filter; this is a low-pass filter, generally used in nuclear imaging.
- The Haar method based on the Haar wavelet coefficients shrinkage corresponding to the original counts, without any preprocessing, using scale-dependent thresholds (see Section 3),
- the Anscombe transform followed by the Bayesian estimator [7],
- the Anscombe transform followed by the Pizurica estimator. Pizurica's estimator is a Bayesian one that consists of estimating the probability that a given wavelet coefficient contains a useful part (i.e., noise-free) called “signal of interest.” It assumes that the coefficients of each subband have a GGD distribution [8].

**Figure 4:**
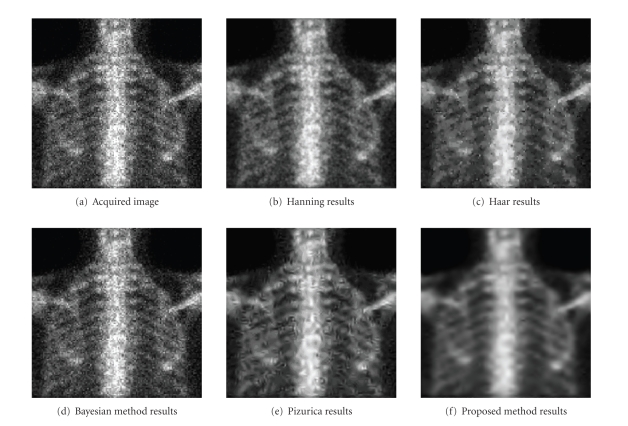
Comparison of the method with several other methods, on a bone scintigraphic image.

**Figure 5:**
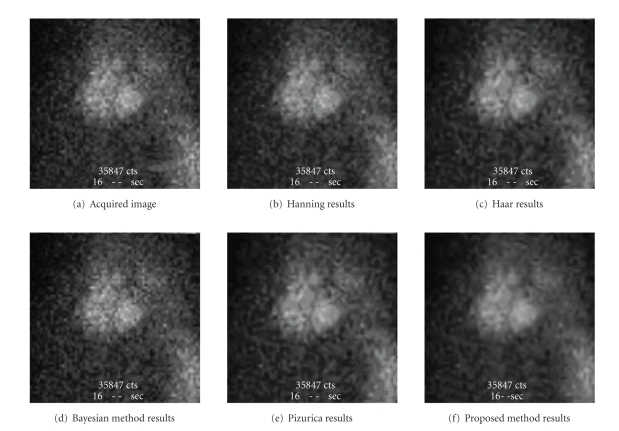
Comparison of the method with several other methods, on a heart scintigraphic image.

Figures 3 and 4 illustrate an example of the obtained results. In Figure 4, we present results obtained by (b) the Hanning method, (c) the Haar method, (d) the Pizurica method (e) the Bayesian method, and (f) the proposed method. Figure 4 corresponds to a bone scintigraphic image, and Figure 5 corresponds to a heart scintigraphic image.

It is clear that the proposed method provides better results than the others. In fact, when other methods fail to remove noise and affect images by artifacts, the proposed method succeeds to remove an important part of noise and to enhance contours.

(2) The second test consists of applying the proposed method to the set of images acquired in an ascendant acquisition time. We can then use objective and subjective evaluation criteria.

### 5.1. Evaluation Using Objective Criteria

The objective criterion consists of computing the Peak Signal to Noise Ratio value (PSNR). The PSNR is calculated between the denoised image acquired with a duration time *T* and the same image acquired in twice this duration time. That is to say that, in each acquisition time, the image with superior duration is considered as the reference one.

**Figure 6:**
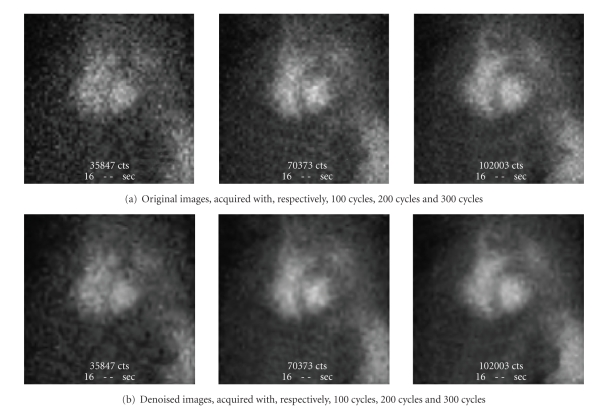
Example of denoising images acquired by ascendant time.

In Figure 6, we present an example of results obtained by denoising images of the heart acquired in an ascendant acquisition time.

In Table 2, we present the computed PSNR, with a sequence of 16 images. In this table,

- Orig.*i*-*t* indicates the original image, *i* indicates the index of the image in the sequence, and *t* indicates the total number of cycles (*t* = 100, 200, or 300);
- Den.*i*-*t* indicates the denoised image, *i* indicates the index of the image in the sequence, and *t* indicates the total number of cycles (*t* = 100, 200, or 300).

Table 2 shows that the benefit obtained for the images of ventriculography acquired with 100 cycles is significant and varies in the interval (0.58–0.74). We can estimate then that the method which we have proposed allowed us to reduce the acquisition time of scintigraphic images. This observation will be confirmed by a subjective evaluation test.

### 5.2. Evaluation Using Subjective Criteria

The subjective criteria consist of using two types of psychovisual tests in collaboration with two nuclear physicians; tests of forced choice and comparative tests [19].

#### 5.2.1. Tests of Forced Choice

These tests compare several images with the original one. The test proposes to the physician two images to be compared to the original one presented in the middle.

**Figure 7:**
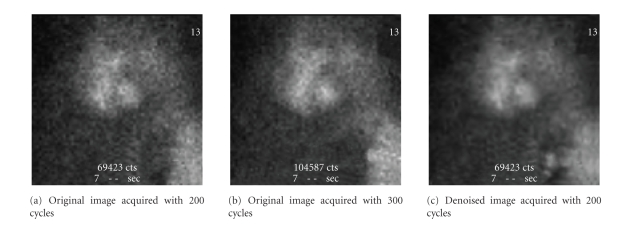
Example of a slide of comparative tests.

In our situation, the two images located on the left and on the right are those acquired with acquisition time *τ* and its denoised version. In the middle, the image of higher acquisition time is placed and considered as reference. The physician is suggested to choose the image which he considers the nearest to the image in the middle. An example of this slide is presented in Figure 7. We note that

- the total counting number is masked in order not to influence the physician choice;
- the images are presented randomly in order to avoid the physician practice to a particular position.

Table 3 presents statistics of the obtained results on a set of 40 images. As shown in this table, the method succeeded to enhance quality of the scintigraphic images, and it made them closer to those acquired with higher duration. Indeed, we note that physicians prefer (with more than 70%) the denoised images than the original ones. This percentage exceeds in certain cases 80%. In a few cases, Physicians do not make any choice (6.25%).

#### 5.2.2. Comparative Tests

**Figure 8:**
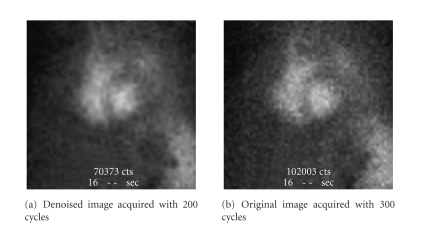
Example of a slide of forced tests.

In these tests, the physician chooses the best images. In our case, the two presented images are the denoised image acquired with a duration *T*, and the same image acquired with a higher duration. An example of this slide is presented in Figure 8. Thus, Table 4 presents statistics of the obtained results on a set of 40 images.

According to these results, physicians prefer, with a percentage higher than 31%, denoised images acquired with 100 cycles to the same images acquired with 200 cycles. This percentage is more important when increasing the counting. Indeed, in more than 59%, the physicians preferred the denoised images acquired with 200 cycles to those acquired with 300 cycles.

In summary, we notice that the proposed method

- improves the PSNR, and preserves the smooth regions, the edges, and the object texture. Denoised images show noise level reduction without loss in contours and images details. On the contrary, other method emphases of Gibbs phenomenon on object boundaries, suppress object edges and textures and affect denoised images by artifacts;
- improves images quality and enhances their texture. In fact, in a given count level, denoised images are quite similar to images at a superior count level. This proves that the proposed procedure can increase the nuclear image quality without increasing the acquisition time and the injected radiopharmaceutical dose.

## 6. Conclusion

In this paper, we presented a novel framework for scintigraphic images denoising. This framework takes benefits from the powerfulness of Bayesian denoising formalism and the Anscombe transform. This framework is based on a modeling step that aims to find the best value of the shape factor in the GGD which models the wavelet coefficients. The shape factor variation is used to compute an adaptive threshold.

Obtained results proved the performance of the proposed method. Indeed, not only the method succeeded to improve nuclear images quality, by reducing counting statistics fluctuations, but also it enhanced their quality and made them closer to the same images acquired with higher acquisition time. This observation can revolutionize the daily nuclear practice. Indeed, in addition to its current use to reduce degradations, image processing can be used to optimize the acquisition time and the amount of the injected radioactive substance.

We note also that this method can be extended to create a general framework for scintigraphic image restoration [20].

## Figures and Tables

**Table 1:** Statistics on the value of *β* (for 100 scintigraphic images).

*β* ≤ 0.5	0.5 ≤ *β* ≺ 1	1 ≤ *β* ≺ 1.5	1.5 ≤ *β* ≺ 2	*β* ≻ 2
3,75%	41,25%	52,92%	1,98%	0,1%

**Table 2:** Statistics on the value of *β* (for 80 scintigraphic images).

	PSNR	PSNR		
	Orig.*i*–100 and	Trait.*i*–100 and	Profit in PSNR	Profit in PSNR
	Orig.*i*–200	Orig.*i*–200	(in db)	(in %)
Orig.1–100	18,63	19,21	0,58	3,09%
Orig.2–100	20,55	21,30	0,74	3,62%
Orig.3–100	20,73	21,38	0,64	3,10%
Orig.4–100	19,59	20,24	0,65	3,34%
Orig.5–100	20,17	20,87	0,70	3,49%
Orig.6–100	19,69	20,40	0,71	3,61%
Orig.7–100	20,60	21,26	0,66	3,22%
Orig.8–100	19,73	20,28	0,55	2,79%
Orig.9–100	19,98	20,62	0,64	3,21%
Orig.10–100	20,59	21,25	0,66	3,19%
Orig.11–100	20,11	20,80	0,69	3,45%
Orig.12–100	19,66	20,28	0,62	3,17%
Orig.13–100	20,41	21,10	0,69	3,39%
Orig.14–100	20,55	21,19	0,64	3,12%
Orig.15–100	19,75	20,37	0,63	3,17%
Orig.16–100	20,15	20,85	0,70	3,45%

**Table 3:** Example of statistics obtained by tests of forced choice.

	Physician1	Physician 2	Average
Original images (100 cycles)	18,75%	25,00%	21,88%
Denoised images (100 cycles)	81,25%	62,50%	71,88%
Not specified	0,00%	12,50%	6,25%

**(a):** 

	Physician 1	Physician 2	Average
Original images (200 cycles)	75,00%	62,50%	68,75%
Denoised images (100 cycles)	25,00%	37,50%	31,25%

**(b):** 

	Physician 1	Physician 2	Average
Original images (300 cycles)	62,50%	18,75%	40,63%
Denoised images (200 cycles)	37,50%	81,25%	59,38%
